# Follow-up imaging of breast symptomatic patients: a waste of radiologist time?

**DOI:** 10.1186/bcr3806

**Published:** 2015-11-05

**Authors:** Diana Dalgliesh, Alison Smith, Anjum Mahatma

**Affiliations:** 1 Royal United Hospital, Bath, UK

## Introduction

The NHSBSP does not recommend early recall following assessment of screen-detected abnormalities. Symptomatic patients in our breast clinic may be invited to return for repeat imaging. A survey of repeat imaging in our symptomatic breast clinic was undertaken to understand whether we can justify reducing the number of patients recalled and to gauge associated anxiety levels.

## Methods

We identified 71 consecutive patients attending an imaging appointment from 1 February 2013 who had a repeat imaging recommendation. Patients were asked to complete a questionnaire. We recorded reason for recall, imaging interval, imaging outcome, and feedback from questionnaires.

## Results

One patient did not attend. Mean interval between initial and repeat imaging: 4−16 weeks. Fifty-five episodes classified R1/R2 at initial imaging; 11 R3; four R4. Outcomes: 68 % were discharged; 11 % were invited for a third imaging appointment and all were then discharged; 13 % had a benign biopsy; 7 % returned to the surgical clinic for management of their benign symptom. Twenty-three questionnaires were completed − one patient was 'very anxious' about repeat imaging, seven patients were 'mildly anxious', 10 were 'relieved', six were 'not bothered'.

## Conclusion

Repeat imaging did not yield any diagnoses of malignancy. All patients were eventually discharged with a benign outcome. We can justify reducing follow-up imaging of our symptomatic patients in line with guidelines for screening assessments. Radiologist time may be better directed towards meeting the symptomatic breast 2-week wait standard.

